# Non-invasive treatment of *Clostridioides difficile* infection with a human-origin probiotic cocktail through gut microbiome-gut metabolome modulations

**DOI:** 10.3389/fmicb.2025.1555220

**Published:** 2025-02-26

**Authors:** Bijay Gurung, Maria C. Courreges, Julie Pollak, Ramiro Malgor, Lin Jiang, Bo Wang, Shaohua Wang

**Affiliations:** ^1^Department of Biomedical Sciences, Ohio University Heritage College of Osteopathic Medicine, Ohio University, Athens, OH, United States; ^2^Infectious and Tropical Disease Institute, Ohio University, Athens, OH, United States; ^3^Department of Chemistry and Chemical Engineering, Florida Institute of Technology, Melbourne, FL, United States; ^4^Division of Natural Sciences, New College of Florida, Sarasota, FL, United States

**Keywords:** hospital-associated diarrhea, *Clostridioides difficile*, probiotics, gut microbiome, gut metabolites

## Abstract

*Clostridioides difficile* (*C. difficile*) is a leading cause of hospital-associated diarrhea, primarily due to gut dysbiosis following antibiotic use. Probiotics have been found to provide several benefits to hosts via modulation of the gut microbiota and their metabolites. However, till now, no conventional probiotics have been clearly proven to be an effective prophylactic option for CDI prevention. Therefore, more studies on developing specific probiotic candidates targeting CDI and improving diversity of probiotics administrated are needed. In this study, a human-origin highly diverse and highly targeted probiotic cocktail (Pro11) containing 11 various probiotic species was developed against *C. difficile.* Pro11 protected mice against CDI with lower clinical scores and higher survival rates, and inhibited *C. difficile in vivo* with less *C. difficile* burden and toxins production determined in colon. Histological analysis demonstrated that Pro11 strengthened gut barrier, reducing gut permeability (less secreted sCD14 in serum) and gut inflammation. In addition, gut microbiome analysis demonstrated that Pro11 increased gut microbiome diversity and beneficial species. Along with gut microbiome modulation, gut metabolites including butyrate, were significantly increased in the probiotics-fed group. Results from this study highlighted probiotics as a promising CDI therapy as gut microbiota modulators, which will lay the foundation for translating probiotics in mitigating CDI and other intestinal pathogens for clinical use.

## Introduction

*Clostridioides difficile* (*C. difficile*) is a gram-positive, spore-forming obligatory anaerobe that is a leading cause of hospital-associated diarrhea. It is responsible for over 500,000 emergency visits and approximately 29,000 deaths each year in the United States, which results in a substantial burden on healthcare systems and the economy, with an estimated annual treatment cost of $6.3 billion (Lessa et al., 2015; Zhang et al., 2016). Disruption of the normal gut microbiome, generally due to antibiotic usage, promotes CDI, which can cause severe damage to the gut epithelium via the production of toxins TcdA and TcdB (Kordus et al., 2022; Patangia et al., 2022). TcdA and TcdB, inside the cytosol inactivate several GTP-binding proteins like Rho, Rac and Cdc42, via glucosylating the target proteins. The glucosylation of the GTPases leads to the actin condensation, cell rounding, cytokine secretion, and ultimately cell death (Carter et al., 2010; Kordus et al., 2022; Voth and Ballard, 2005). Antibiotic-induced gut dysbiosis reduces microbial metabolites, including secondary bile and short-chain fatty acids, which help protect against CDI (Aguirre and Sorg, 2022; Ouyang et al., 2022). Although antibiotics like vancomycin are commonly used to treat CDI, they also disrupt gut microbiota, increasing the risk of recurrent CDI (rCDI). Between 15 to 30% of the patients who had acquired the CDI developed rCDI, with approximately 40–60% of the patients experiencing additional recurrences (Cornely et al., 2012). Due to these challenges and the emergence of antibiotic-resistant *C. difficile* strains, the Centers for Disease Control and Prevention (CDC) has classified it as an urgent threat, signifying the importance of developing efficient prevention and therapeutic strategies (Solomon and Oliver, 2014).

Current treatment strategies for CDI primarily involve antibiotics like vancomycin or fidaxomicin (Cornely et al., 2012; Leong et al., 2018). However, the risk of recurrent CDI associated with antibiotic use limits the effectiveness of these treatment options. Fecal microbiota transplantation (FMT) is another therapeutic option, but it also has its limitations, such as the potential transfer of harmful pathogens and the lack of standard protocols (Alrabaa et al., 2017; Fischer et al., 2017; Kumar and Fischer, 2020). These limitations call for the exploration of alternative therapeutic approaches. Given the role of gut dysbiosis in CDI pathogenesis, probiotics represent one such alternative, as they may help restore the gut microbiota and protect against *C. difficile* (Allen et al., 2013; Barbosa et al., 2023; Kaewarsar et al., 2023; Wei et al., 2018). Although numerous studies have investigated probiotics for CDI treatment, results have been inconsistent (Allen et al., 2013; Éliás et al., 2023; Heil et al., 2021; Li et al., 2019), and till date, no conventional probiotics have been clearly proven as an effective prophylactic option (European Society of Clinical Microbiology and Infectious Diseases Study Group on *Clostridioides difficile*, ESGCD) and Study Group for Host and Microbiota interaction (ESGHAMI) (Pal et al., 2022). The low efficacy of commercial probiotics in clinical settings may be due to limited diversity in their formula, while the gut microbiome is much more complex, so more research uncovering diverse organisms with potential in treating CDI is necessary (Kalakuntla et al., 2019). Another reason may be the random screening, lacking careful selection of specific organisms using a mechanistic-based approach, leading to less targeting effects on CDI (Pal et al., 2022). Compared to conventional probiotics, which are typically isolated from various food sources, human-originated probiotics consist of commensal microbes naturally found in the human body (Ahmadi et al., 2020; Fontana et al., 2013). Given the growing need for alternative therapeutic strategies against *C. difficile* and the critical role of gut microbiota in inhibiting the CDI, isolating and studying the probiotic strains that are already part of the gut microbiota holds significant potential. Several studies have explored the isolation of human-origin probiotics and their potential benefits to the host including gut microbiota modulation (Ahmadi et al., 2020; Nagpal et al., 2018). Notably, a human origin mix (VE303), a consortium of eight *Clostridia* strains, has shown promise in combating *C. difficile* and is currently in a phase 3 clinical trial for recurrent CDI. However, challenges persist, including risks of toxins release and treatment-emergent adverse events (Louie et al., 2023). Therefore, further research is essential to develop more probiotic candidates targeting CDI and to enhance the diversity of probiotic formulations for optimal therapeutic outcomes.

To address this limitation, we developed a human-origin probiotic cocktail with high CDI-targeting specificity and diversity as a non-invasive treatment for CDI. Our *in vivo* and *in vitro* studies demonstrated how the probiotic cocktail protected mice against CDI by modulating the gut microbiota and gut metabolome. These results provide new perspectives for developing effective probiotics as a therapy for CDI and establish a strong foundation for comprehensive studies to translate this approach into clinical use.

## Materials and methods

### Isolation, identification, and characterization of human-origin probiotics

Baby diapers (unidentified) containing fecal samples were collected from the Child Development Center at Ohio University (Athens, OH). Fecal samples from 27 individual diapers (0.5 g) were resuspended in 5 mL of MRS (for isolation of *Lactobacillus* strains), MRS-L-cysteine (MRS plus 0.1% of L-cysteine for isolation of *Bifidobacterium* strains), and LM17 (M17 supplemented with 2% lactose, for isolation of *Streptococcus* strains). After incubation at 37°C (*Lactobacillus* and *Bifidobacterium* strains) and 42°C (*Streptococcus* strains) for 24 h, cell cultures were serially diluted and spread onto the corresponding selection agar medium plates (MRS for *Lactobacillus*, TOS-propionate for *Bifidobacterium*, and LM17 for *Streptococcus* strains), and cultivated for 12–24 h. At least 10 colonies were picked and purified with the streak plate method. Colony PCR with *Lactobacillus* specific primer pairs (5’-TGGAAACAGRTGCTAATACCG-3′; 5’-GTCCATTGTGGAAGATTCCC-3′) (Byun et al., 2004), *Bifidobacterium* specific primer pair (5′- GGGTGGTAATGCCGGATG-3′; 5’-CCACCGTTACACCGGGAA-3′) (Matsuki et al., 2003), and *Streptococcus thermophilus* specific primer pair (5′- CACTATGCTCAGAATACA-3′; 5’-CGAACAGCATTGATGTTA-3′) (Lick et al., 1996) were performed with colonies from the corresponding screening medium plates. Colonies demonstrating corresponding specific bands were chosen for further identification by sequencing (GeneWiz LLC, NJ, USA) of the 16S rDNA amplified with the 27 F and 1492 R universal primers (27 F: 5′-AGAGTTTGATCCTGGCTCAG-3′ and 1492 R: 5′-GGTTACCTTGTTACGACTT-3′) as being described in our previous study (Nagpal et al., 2018).

### Screening of the most efficient probiotics based on their inhibitory activity against *Clostridioides difficile*

After isolation, probiotic strains were screened for their inhibitory activity against *C. difficile* with soft layer agar diffusion methods described by Karska-Wysocki et al. (2010). The probiotic strains were subcultured into the corresponding broth medium: MRS for *Lactobacillus,* MRS-L-cysteine for *Bifidobacterium*, and LM17 for *Streptococcus.* After incubation at 37°C for 12 h, 5 μL of each probiotic strain were spotted on the MRS agar plates and incubated at 37°C. After 24 h, the spots were overlayed with BHIS (brain heart infusion broth supplemented with 0.5% yeast extract and 0.1% L-cysteine) (Wang et al., 2018) soft agar containing 200 μL of overnight culture of *C. difficile*. Then, the plates were incubated at 37°C for 24 h in an anaerobic chamber (90% N_2_, 5% CO_2_, 5% H_2_ by volume) (Wang et al., 2024), and the zone of inhibition was measured.

### Antibiotic susceptibility test

Disc diffusion tests were performed to determine the antibiotic susceptibility of the probiotic strains with antibiotic discs (BBL™ Sensi-Disc™) from BD Life Sciences, USA. *Lactobacillus, Bifidobacterium*, and *Streptococcus* strains were cultivated overnight in MRS, MRS-L-Cysteine, and LM17, respectively. 100 μL of each fresh culture was mixed with the corresponding soft agar and overlaid onto corresponding agar plates. Once the soft agar solidified, antibiotic discs containing tetracycline (30 μg), penicillin (6 μg), erythromycin (15 μg), novobiocin (30 μg), chloramphenicol (30 μg), and streptomycin (10 μg) were placed on the plates. The plates were incubated overnight at 37°C, and diameters of the inhibition zones were measured in millimeters. Antibiotic susceptibility was categorized as resistant (R), intermediate (I), or susceptible (S) according to Clinical and Laboratory Standard Institute (CLSI) guidelines.

### Co-culture of the probiotic strains and *Clostridioides difficile*

To further validate the inhibitory effect of the selected probiotic strains, co-culture of each probiotic strain with *C. difficile* was performed. Probiotic strains were cultured in their corresponding liquid media, MRS, MRS-L-Cysteine, and LM17 for *Lactobacillus, Bifidobacterium,* and *Streptococcus*, respectively. After overnight anaerobic cultivation, each probiotic strain was inoculated at 1% (v/v) into 5 mL of BHIS medium, with *C. difficile* inoculated at the same ratio. BHIS inoculated with only *C. difficile* (1% v/v) was set as control. After 6 h cultivation at 37°C, the co-culture was serially diluted, and plated on the BHIS plates containing cefoxitin and D-cycloserine for selective detection of *C. difficile* (Dsouza et al., 2022)*. C. difficile* was enumerated after 48 h of anaerobic cultivation at 37°C. The experiments were performed in triplicate and repeated three times.

### Effects of cell-free supernatant (CFS) on biofilm formation of *Clostridioides difficile*

The effect on biofilm formation of *C. difficile* was studied using CFS from the 11 probiotic strains. The cell-free supernatant was obtained by centrifuging (13,000 g, 5 min) the probiotic strains culture (12 h cultivation), followed by filtration through 0. 0.22 μm filters. The CFS was diluted (1:10) with BHISG medium (BHIS supplemented with 0.1 M glucose), where 1% of the overnight *C. difficile* culture was inoculated, and cultivated in 96 well tissue culture-treated polystyrene plates anaerobically at 37°C for 24 h. Optical density (OD) was measured at 600 nm to assess the growth using a Synergy H1 microplate reader (BioTech). The CV staining method was used for biofilm assay (Dawson et al., 2012; Willett et al., 2019), where planktonic bacteria were removed, and the wells were washed with phosphate buffer saline (PBS), stained with crystal violet (CV), and dissolved in methanol to read the absorbance at 595 nm. The effects of probiotics CFSs on biofilm formation were normalized by calculating the ratio of biofilm-specific staining to the overall cell density.

### Preparation of probiotic cocktail

The strains exhibiting the highest growth inhibition against *C. difficile* in each species including five *Bifidobacterium*, four *Lactobacillus*, and two *Streptococcus* strains, were selected to formulate a probiotic cocktail containing 11 strains. To prepare the probiotic cocktail for mice study, individual probiotic strains were cultured in their respective media (MRS for *Lactobacillus*, MRS-L-Cysteine for *Bifidobacterium*, and LM17 for *Streptococcus*). Cultures were harvested during the later logarithmic growth phase, combined in a 1:1 ratio based on optical density (OD 600 nm), washed twice with PBS, resuspended in 1/10 of the total volume (relative to the combined volume of all 11 strains) in PBS with glycerol, and stored at −80°C. The final concentration of the cocktail was quantified using MRS agar under anaerobic conditions.

### Mouse model of infection

Spores of *C. difficile* were prepared as described by Theriot et al. by cultivating *C. difficile* (ATCC43255) with Clospore media at 37°C anaerobically for 5–7 days (Perez et al., 2011; Theriot et al., 2016). Spores were heat-treated for 20 min at 65°C to kill the remaining vegetative bacilli and enumerated by cultivation on TCCFA (taurocholate, cefoxitin, cycloserine, and fructose agar) plates. Mice from the probiotics group were given probiotic cocktail (5 × 10^9^ CFU/mL) in drinking water (1 mL of probiotic cocktail at 10^12^ CFU/mL was added to 200 mL of the drinking water) starting one week before antibiotic treatment and continuously throughout the whole process (Ahmadi et al., 2020). Mice from the control group were given the same amount of glycerol in drinking water. The special drinking water was changed every 2 days. C57BL/6 wild-type male mice (6–8 weeks old) were given antibiotic (cefoperazone, 0.5 mg/mL) in sterile drinking water for 5 days with changes every 2 days, followed by 2 days regular drinking water before challenge with *C. difficile* spores (100 μL of 10^6^ CFU, delivering 10^5^ CFU per mice) through oral gavage (Fletcher et al., 2018). The mice were monitored for 7 days, and clinical scores including body weight, activity, posture, eyes, coat, diarrhea, and dehydration were recorded. Mice were monitored every 6 hours during the daytime from day 1 to 3 post-infection, then once daily until 7 days post-infection. Disease severity was assessed using a scoring system (0: normal, 1: mild, 2: moderate; 3: severe), with the total score calculated as the sum of all recorded signs. Mice were euthanized if their clinical score exceeded 12. All the animal studies were conducted following procedures approved by Ohio University Heritage College of Osteopathic Medicine, Animal Research Program’s Institutional Animal Care and Use Committee (IACUC).

### *Clostridioides difficile* burden and toxins production

Fecal samples collected one day post infection were weighed and vortexed in PBS (1 mg feces/10 μL PBS). After settling down for 10 min, supernatants were diluted serially, and *C. difficile* colonies were enumerated on TCCFA plates after 2–3 days of anaerobic cultivation. Concentration of *C. difficile* toxins (TcdA/B) in fecal samples was measured with Fecal *C. difficile* Toxin A & B ELISA Kit from EDI (Epitope Diagnostics, INC.) according to the instruction.

### Gut leakage markers determination with enzyme-linked immunosorbent assay (ELISA)

Mice serum samples were collected after 2 days of infection, and the concentration of secreted CD14 and LBP were tested by Mouse CD14 Quantikine ELISA Kit (R and D Systems) and Mouse LBP PicoKine ELISA Kit (Boster Bio) following the protocols provided by suppliers. Results were read with a Synergy H1 microplate reader (BioTech) plate reader.

### Histological analyses

After 2 days of infection, intestine tissues (colon) were harvested, washed with PBS, fixed in 10% formalin overnight, and paraffin embedded for histological assays. Sections (0.5 μm thickness) were stained with hematoxylin and eosin (H&E) and imaged with an AmScope microscope on ×10 magnification at the Histology Core Facility at Ohio University.

### Gut microbiota analysis

Gut microbiota composition was compared according to protocols described previously (Nagpal et al., 2018; Wang et al., 2019). Briefly, genomic DNA was extracted from ~100 mg of mice feces using the QIAamp Fast DNA Stool Mini Kit (QIAGEN). The V4 region of bacterial 16S rDNA was amplified and sequenced with an Illumina P1 600cyc NextSeq2000 Flowcell platform at SeqCenter (Pittsburgh, PA). The sequences were de-multiplexed, quality filtered, clustered, and analyzed with Quantitative Insights into Microbial Ecology (QIIME) and R-based analytical tools. Linear discriminatory analysis (LDA) effect size (LefSe), a statistical method that analyzes the relative abundance of the bacterial taxa in the samples (Wang et al., 2022), was used to identify unique bacterial taxa driving differences after probiotic treatment.

### Gut metabolomics analysis

Fecal metabolites were analyzed with NMR. Fecal samples collected after one day infection were resuspended in water, followed by sonication and centrifugation (12,000 g, 10 min, 4°C) to obtain soluble fractions, followed by being mixed with PBS (pH 7.4) containing 10% D_2_O and 0.1 mM trimethylsilyl propionate (TSP). NMR experiments were performed with a Joel 400 MHz NMR using a presaturation experiment on Delta 5.3.3 (JEOL Ltd.) with 64 scans and 4 s relaxation delay. NMR spectra were preprocessed with zero-filled 1 time and windows function single exponential 0.25 Hz and transferred to Matlab for data analysis. NMR peak intensities were obtained using the average peak width approach previously reported with slight adjustment (Wang et al., 2020), and total intensity normalization was applied before further data analysis. The metabolite identification was carried out using Chenomx 8.6 (Chenomx Inc.).

### Statistical information

Statistical differences among groups/treatments were analyzed using two-tailed unpaired Student’s *t*-test and/or ANOVA. All the assays were performed at least two to three times with three to five replicates at each time and *n* ≥ 5 animals in each group, and the values presented in graphs/tables are means ± standard error of means or means ± SEM. Principal component analysis (PCA) created in R statistical software package was applied to distinguish microbiota and metabolism features of *C. difficile* with and without probiotics treatment. LefSe was used to identify unique bacterial taxa that drives differences in probiotics-treated samples and control. GraphPad (Prism9) was used for making figures. *p* < 0.05 was considered statistically significant.

## Results

### Isolation, identification, and screening probiotic strains against *Clostridioides difficile*

**Figure 1:**
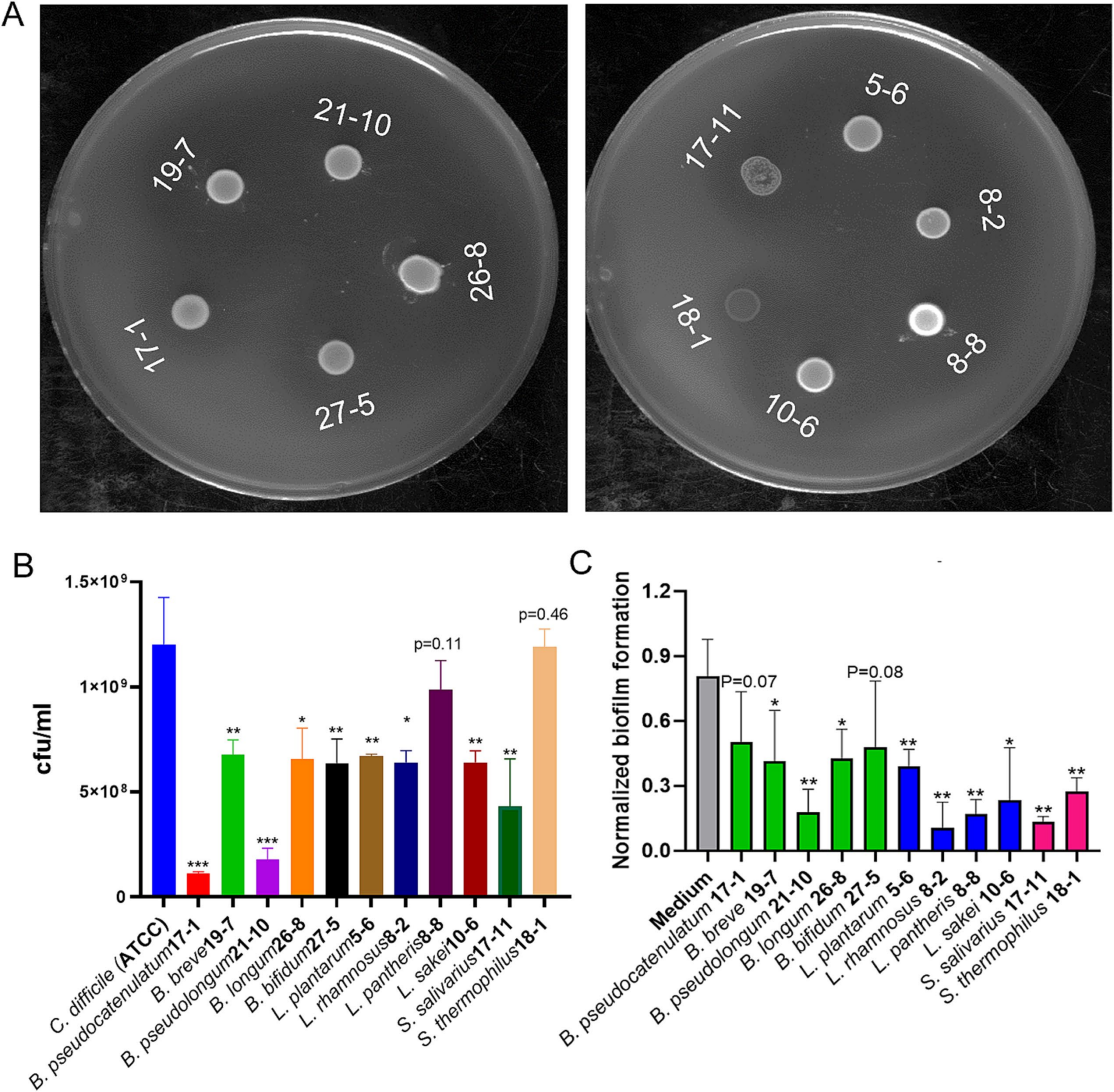
Effects of probiotics on growth and biofilm formation of *C. difficile*. **(A)** Probiotic strains inhibited growth of *C. difficile* on double layer soft layer with inhibition zones around cultures. **(B)** Nine of the 11 probiotic strains showed significant growth inhibition against *C. difficile* during co-culture in broth (Data represent mean ± SE from three independent experiments, statistical significance was analyzed using a two-tailed Student’s *T*-test). **(C)** Cell free supernatant (CFS) of all the 11 probiotics reduced biofilm formation of *C. difficile* (Data represent mean ± SE from three independent experiment, statistical significance was analyzed using a two-tailed Student’s *t*-test). *p*-values for differences between the co-culture and the single *C. difficile* culture control, *p < 0.05, **p < 0.01, and ***p < 0.001.

Probiotic strains from the *Bifidobacterium, Lactobacillus,* and *Streptococcus* genus were isolated from stool samples from infant diapers. Colony PCR with genus-specific primers (Table 1) was performed to identify the genus of the isolates, followed by being further identified using 16S rRNA gene sequencing. Figure 1A shows the results of the soft-layer agar diffusion assay, where the circular spots represent individual probiotic strains, and the lawn is from *C. difficile*. Based on their effects on inhibiting the growth of *C. difficile* (the inhibition zone in Figure 1A) and their sensitivity to commonly used antibiotics (Supplementary Figure S1), the top probiotic strain demonstrating the largest inhibition zone against *C. difficile* and the greatest sensitivity to antibiotics from the same species was selected. A cocktail consisting of 11 isolated strains (4 *Lactobacillus*, 5 *Bifidobacterium*, and 2 *Streptococcus* strains) was developed and named Pro11.

**Table 1:** List of probiotic strains used for the study.

ID	Identification	Name used in manuscript	Accession number in NCBI
BSW 17–1	*B. pseudocatenulatum*	*B. pseudocatenulatum* 17–1	PQ454214
BSW 19–7	*B. breve*	*B. breve 19–7*	PQ454215
BSW 21–10	*B. pseudolongum*	*B. pseudolongum* 21–10	PQ454216
BSW 26–8	*B. longum*	*B. longum* 26–8	PQ454217
BSW 27–5	*B. bifidum*	*B. bifidum* 27–5	PQ454218
LSW 5–6	*L. plantarum*	*L. plantarum* 5–6	PQ454208
LSW 8–2	*L. rhamnosus*	*L. rhamnosus* 8–2	PQ454209
LSW 8–8	*L. pantheris*	*L. pantheris* 8–8	PQ454210
LSW 10–6	*L. sakei*	*L. sakei* 10–6	PQ454211
SW 17–11	*S. salivarius*	*S. salivarius* 17–11	PQ454212
SW 18–1	*S. thermophilus*	*S. thermophilus* 18–1	PQ454213

To further assess the inhibitory activity of the selected probiotic strains against *C. difficile*, a co-culture test with *C. difficile* and each of the probiotic strain in broth was performed. Nine of the 11 individual probiotic strains showed significant inhibition against growth of *C. difficile*. While the other two strains, *L. pantheris* 8–8 and *S. thermophilus* 18–1, slightly inhibited growth of *C. difficile* (Figure 1B). The strain with the highest inhibitory activity was shown by *B. pseudocatenulatum*, which reduced *C. difficile* to less than 10% of the control level (Figure 1B). Besides co-culture of individual probiotics directly with *C. difficile*, cell free supernatant (CFS) of probiotics were also tested for their effects on biofilm formation of *C. difficile*, one of the critical pathogenic factors, responsible for antibiotics-resistance and recurrent CDI (Vuotto et al., 2018). As shown in Figure 1C, CFS from all the 11 probiotic strains demonstrated significant inhibition of biofilm formation of *C. difficile*.

### The probiotic cocktail protected mice against CDI

**Figure 2:**
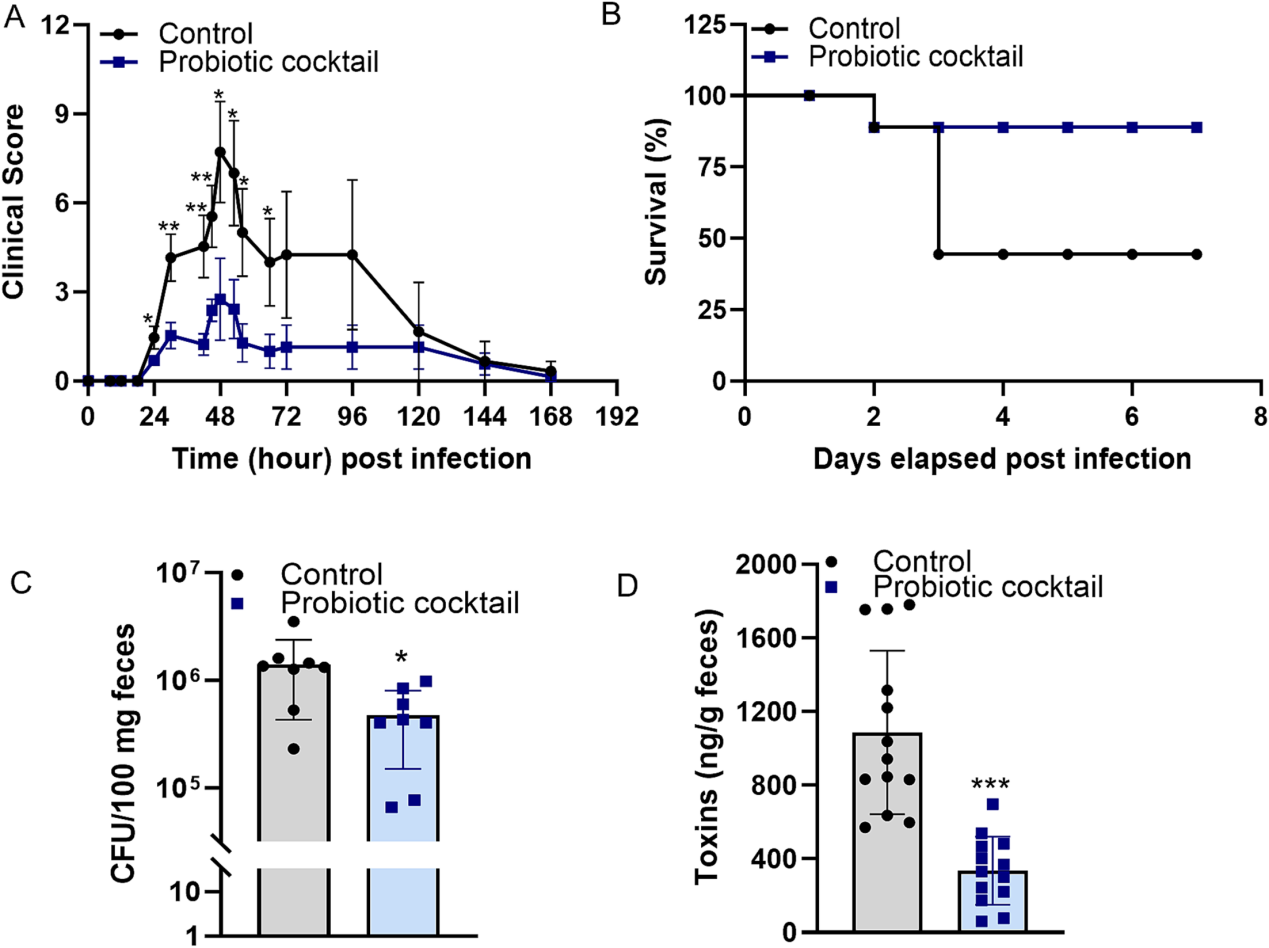
Probiotic cocktail protected the host during *C. difficile* infection (CDI). Probiotic cocktail ameliorated CDI in mice (*n* = 8 per group) with lower clinical scores **(A)** and higher survival **(B)** compared to that from the control group [Panel **(A)**: data represent mean ± SE from two independent experiments. Statistical significance was analyzed using a two-tailed Student’s T-test for different time points]. Analysis with fecal samples indicated roles of the probiotics cocktail in reducing burden **(C)** and toxins production of *C. difficile* **(D)** *in vivo*. [Panel **(C)**: data represent mean ± SE from *n* = 8 mice. Panel **(D)**: data represent mean ± SE from *n* = 13 mice. Statistical significance was analyzed using a two-tailed Student’s t-test]. *p*-values for differences between the probiotics-fed and control mice, *p < 0.05, **p < 0.01 and ***p < 0.001.

Clinical scores were monitored during *C. difficile* infection (Figure 2A). Peak clinical scores were observed on day two post infection from both control and Pro11-fed group. However, the clinical scores in the Pro11-fed group demonstrated significantly lower scores than the control group, indicating the protection of probiotic cocktail against *C. difficile* infection. In terms of survivability, which is another measure of disease severity, our findings indicate that the probiotic cocktail enhanced the survival rate to 80%, compared to that (40%) from the control mice (Figure 2B). Fecal samples collected one day post infection were used to determine *C. difficile* burden and toxin concentration. Pro11-fed mice had significantly lower fecal *C. difficile* burden (33.7% of that from control group) (Figure 2C) and lower fecal toxin concentration (30.9% of that from control group) (Figure 2D). The lower clinical scores, higher survival rates, reduced *C. difficile* burden and toxins demonstrated by the Pro11-fed mice indicated that our probiotic cocktail was able to provide protection and reduce the symptoms associated with CDI *in vivo*.

### The probiotic cocktail reduced gut permeability and gut inflammation

**Figure 3:**
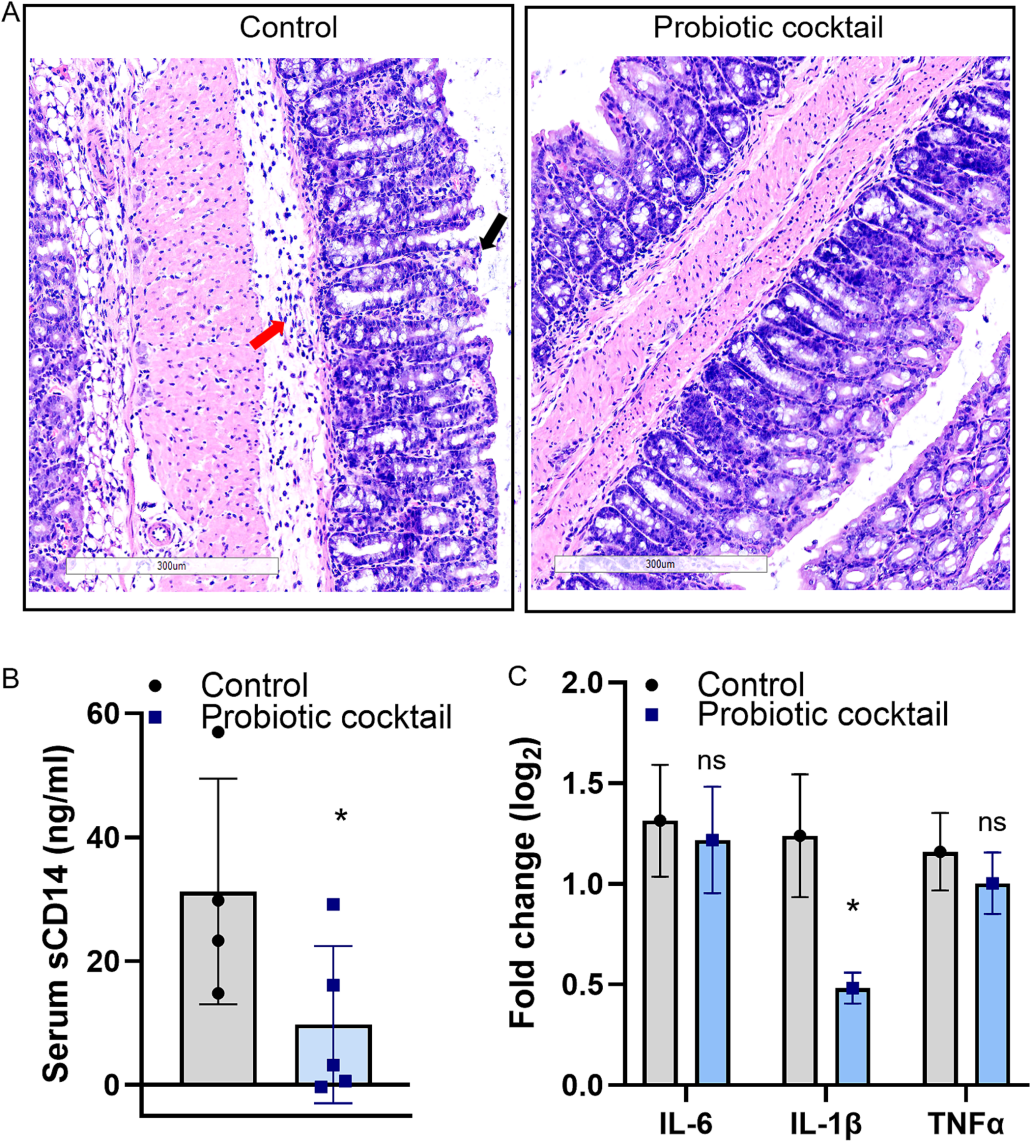
Probiotic cocktail strengthened gut barriers and reduced inflammation during CDI. **(A)** Histopathological analysis of the colon tissue using hematoxylin and eosin (H&E). Comparing to the increased inflitration of neutrophils (red arrow) and disruption of the villi (black arrow) in control due to CDI, the probiotic cocktail-fed mice maintained comparable intact villi and less inflitration of neutrophils. Accordingly, less serum sCD14 concentration **(B)** and reduced pro-inflammatory marker IL-1β **(C)** were detected in the probiotics group [Panel **(B)**: data represent mean ± SE from *n* = 4 mice serum. Panel **(C)**: data represent mean ± SE from *n* = 4 mice colon tissues. Statistical significance was analyzed using a two-tailed Student’s t-test]. *p*-values for differences between the probiotics-fed and control group, *p < 0.05, ns, non-significant difference.

Histological analyses for colon tissues with H&E staining are shown in Figure 3. Compared to the disrupted mucus layer and broken villi in the control mice, the mucus layer and villi structure from Pro11-fed mice appears to remain intact (Figure 3A). Moreover, along with the comparable intact mucus layer, significantly lower sCD14 was determined in the serum of the Pro11-fed mice (Figure 3B), indicating that probiotics ameliorate CDI-induced gut permeability with less sCD14 secreted from gut to serum. Additionally, in the control group, the tissue showed signs of inflammation and increased inflammatory cells (Figure 3A). To further study the role of probiotic strains in reducing inflammation, we measured the expression of pro-inflammatory markers in the colon tissues using a RT-PCR. Although no significant differences in expression of IL-6 and TNF-*α* were observed, IL-1β was significantly decreased in the probiotics-fed mice compared to the control (Figure 3C), suggesting effects of the probiotic cocktail on reducing inflammation during CDI. These results suggested that the probiotic cocktail could maintain comparable strengthened gut barriers, resulting in reduced gut permeability and gut inflammation.

### The probiotic cocktail ameliorated CDI through beneficially modulating gut microbiome-gut metabolome

**Figure 4:**
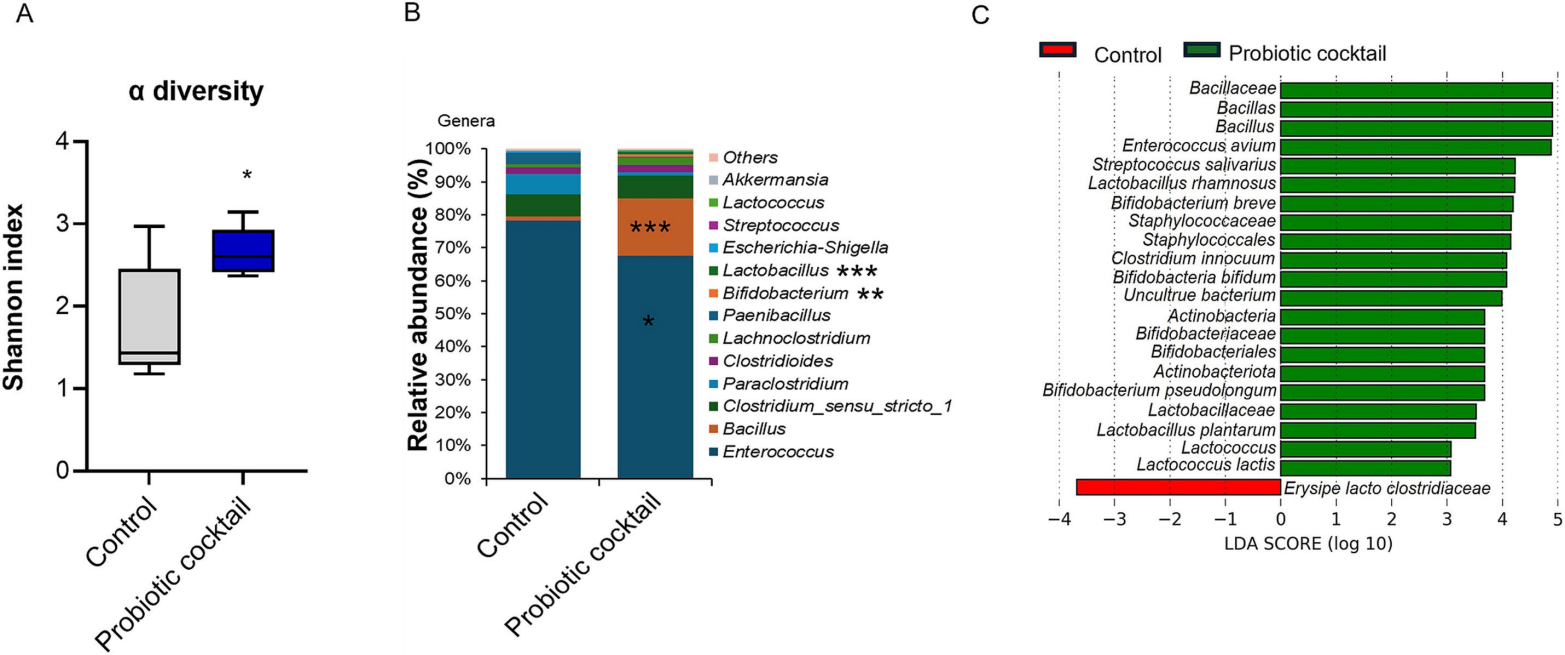
Probiotic cocktail beneficially modulated gut microbiome during CDI. Probiotics prompted gut microbiome diversity **(A)**, enhanced beneficial genus *Lactobacillus, Bifidobacterium* and *Bacillus*, while reducing the *Enterococcous* genus **(B)** compared to the control (without Pro11 treatment). **(C)** Linear discriminatory analysis (LDA) effect size (LefSe) demonstrated representative species significantly modulated with probiotics treatment. *p*-values for differences between the probiotics-fed and control mice, *p < 0.05, **p < 0.01 and ***p < 0.001.

As gut dysbiosis is one of the primary factors in enhancing CDI pathogenesis, modulation of the gut microbiota will be a promising strategy for CDI therapy (Feuerstadt et al., 2022; Gonzales-Luna et al., 2023; Piccioni et al., 2022). Probiotics have been reported about their roles in modulating gut microbiota via increasing the diversity and population of the beneficial gut microbiota (Barathikannan et al., 2019; Bloemendaal et al., 2021; Muwonge et al., 2021). To explore the involvement of Pro11 in gut microbiota modulation and protection against CDI, gut microbiome analysis with fecal samples collected one day post *C. difficile* infection were performed. As shown in Figure 4A, higher alpha diversity was demonstrated by the Pro11-fed mice. Significant differences were found on the phyla level, with decreased Firmicutes and increased Actinobacteria after probiotics treatment during CDI (Supplementary Figure S2). Furthermore, linear discriminant analysis (LDA) effect size (LEfSe) demonstrated that the families *Bifidobacteriaceae, Lactobacillaceae,* and *Bacillaceae* were enriched in the Pro11-fed group, whereas *Erysipelatoclostridaceae* was enriched in the control group (Supplementary Figure S2). On the genus level, as expected, beneficial bacteria from the genus *Lactobacillus, Bifidobacterium*, and *Bacillus* significantly increased, and *Enterococcus* which contains lots of potential pathogens decreased in the Pro11-fed group (Figure 4B). On the species level, the increase of beneficial species in the Pro11-fed mice includes the *Lactobacillus*, *Bifidobacterium*, *Streptococcus* species which our probiotic cocktail belong to, as well as other probiotics from *Lactococcus* and *Enterococcus* species (Figure 4C). These results suggest that the newly developed probiotic cocktail can ameliorate gut microbiome dysbiosis by increasing the diversity and the relative abundance of the microbiome that can exert beneficial effects on host along with the protection against *C. difficile.*

**Figure 5:**
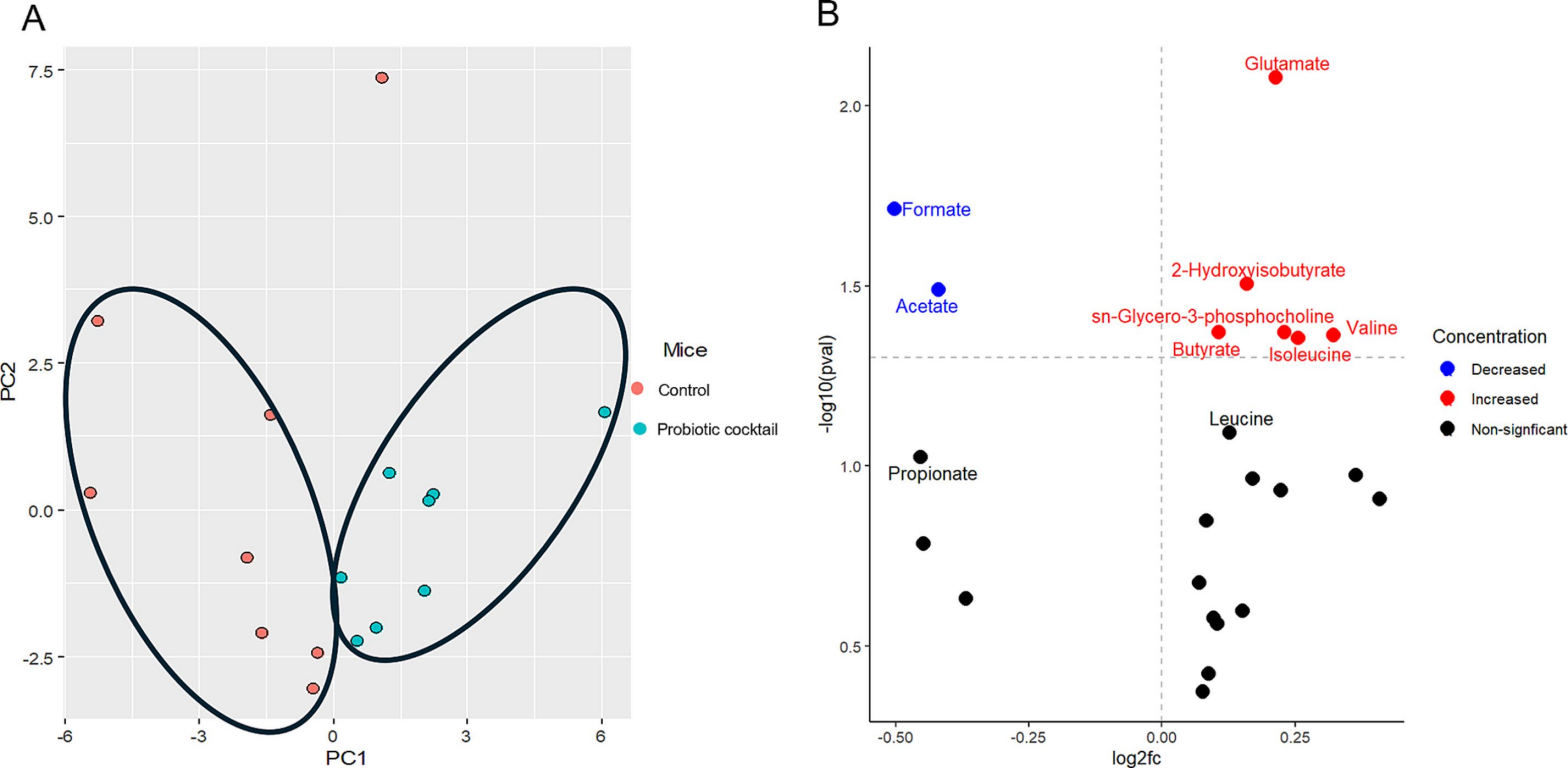
Probiotic cocktail modulated gut metabolites. **(A)** PCA plot demonstrated significant metabolites clusters for the control and probiotic cocktail groups. The PC1 and PC2 in **(A)** represent the first and second principal components and are useful to visualize the differences between the groups. **(B)** Volcano plot illustrated the specific metabolites significantly increased (red), decreased (blue), and nonsignificant (black) modulated in the probiotics group compared to the control during CDI.

Along with the modulation of the gut microbiome, changes in the gut microbial metabolites were also compared using the global metabolomics analysis between the two groups. As expected, principal component analysis (PCA) revealed that gut metabolites from Pro11-fed mice clustered in different regions compared to those from the control group, indicating modulation of gut metabolites by Pro 11 treatment (Figure 5A). Significantly increased metabolites in the Pro11-fed group includes 2-hydroxyisobutyrate, butyrate, glutamate, Sn-glycero-3-phosphcoline, leucine, and valine. While formate and acetate were found to be decreased with probiotics treatment (Figure 5B). These results suggest that probiotics feeding led to changes in gut metabolites, which could potentially contribute to the prevention against *C. difficile*.

## Discussion

*Clostridioides difficile* infection is one of the leading causes of hospital-associated diarrhea (Bien et al., 2013; Matzaras et al., 2022). Disruption of the normal gut microbiota due to antibiotic usage is a major risk factor for CDI (Finn et al., 2021; Patangia et al., 2022; Schäffler and Breitrück, 2018). Vancomycin, a primary therapeutic agent, is commonly used to treat CDI. However, it can exacerbate the condition by disrupting the gut microbiota, increasing the risk of recurrent CDI (Isaac et al., 2017). This highlights the need for alternative therapeutic strategies that not only protect against *C. difficile* but also restore gut microbiota homeostasis and metabolite balance. One such therapeutic strategy that has been used successfully against CDI is the fecal microbiota transplantation (FMT), which works by restoring gut microbiota and providing protection against CDI, but the limitation of FMT includes the potential risk of transfer of pathogens (Pamer, 2014). These issues, combined with the substantial burden of CDI on patients and healthcare systems, underscore the urgency of developing new treatment strategies. Therefore, probiotics, which modulate the gut microbiota, have emerged as a potential strategy for protecting against CDI (Barbosa et al., 2023; Gaisawat et al., 2020; Kaewarsar et al., 2023).

Probiotics are known to benefit the host and can help inhibit specific pathogens (Martin-Gallausiaux et al., 2021; Wei et al., 2018), including *C. difficile*, via colonization resistance and by producing bacteriocins, metabolites, and compounds that makes the gut environment unfavorable for pathogenic growth (Arakawa et al., 2008; Hayashi et al., 2021; Schubert et al., 2015; Theriot et al., 2016). While probiotics have been used to treat various conditions, their use in CDI treatment has yielded mixed results. For example, a study by Allen et al. (2013) showed that the administration of mixture of three strains (*L. acidophilus, B. bifidum* and *B. lactis* strains) in older people did not provide protection against *C. difficile* (Allen et al., 2013). Whereas a study by Hudson et al. (2019) showed that the use of probiotics (*L. acidophilus* and *S. boulardii*) by the patients taking broad spectrum antibiotics helped in reducing the incidence of CDI compared to the patients who did not receive the probiotics (Hudson et al., 2019). To date, no conventional probiotics have been clearly proven to be an effective prophylactic option for CDI prevention (Pal et al., 2022), which may be due to lacking of diversity in their formula, and/or lacking careful selection of specific organisms. Therefore, more studies on developing specific probiotic candidates targeting CDI and improving diversity of probiotics administrated are needed.

In this study, we screened probiotics from human stool samples for their effects on inhibiting the growth of *C. difficile*. For our study, we focused on isolation of the probiotic strains from the genus *Lactobacillus, Bifidobacteria,* and *Streptococcus*, which comprise of bacterial species that have been widely used as probiotics. And previous studies have investigated these probiotic genera against *C. difficile,* with mechanisms such as bacteriocin production, lactic acid secretion, and colonization resistance (Jo et al., 2023; Kolling et al., 2012; Lee et al., 2021; Spinler et al., 2017). To increase the diversity of the probiotic cocktail, the top strains from each species with the highest inhibition activity against *C. difficile* were selected to develop a new probiotic cocktail containing 11 different strains. Notably, from soft agar overlay experiments (where *S. salivarius* and *S. thermophilus* demonstrated lower inhibition) and co-culture experiments (where *L. pantheris* and *S. thermophilus* demonstrated lower inhibition) (Figure 1A). Despite their comparatively lower inhibition, these strains were included in the cocktail for their potential benefits (Kaci et al., 2014; Kolling et al., 2012) and to increase the diversity of the probiotic cocktail. Co-culture experiments further verified their inhibition effects, with *L. pantheris* and *S. thermophilus* showing slight inhibition and all the other probiotic strains showed significant inhibition of *C. difficile* growth (Figure 1B). Interactions and competition for resources between pathogens and probiotics in a natural environment can play a major role in inhibiting *C. difficile*. Therefore, co-culture experiments can provide valuable insights into the interactions between probiotic strains and *C. difficile*, as well as their effects on *C. difficile* growth. While the current co-culture study focuses on growth inhibition, ongoing research aims to further investigate the underlying mechanisms and interactions between probiotic strains and *C. difficile*. Interestingly, all the CFS showed inhibitory action against the biofilm formation of *C. difficile* (Figure 1C). Biofilm formation is a significant factor contributing to CDI pathogenesis (Wang et al., 2024), as biofilms have higher concentrations of sessile cells and are more resistant to treatments than planktonic cells (Costerton, 2007), playing prominent roles in CDI recurrence and antibiotic resistance (Taggart et al., 2021). The inhibitory effects of the CFS on biofilm formation suggest that these probiotics could serve as an effective strategy to disrupt biofilms, potentially improving treatment outcomes and reducing relapse rates. Referring to potential mechanisms under probiotics’ *in vitro* inhibition effects against *C. difficile*, metabolites produced by probiotics such as organic acids (Jo et al., 2023; Tejero-Sariñena et al., 2012) and bacteriocins (Todorov et al., 2020) have been suggested to play roles in providing protection against *C. difficile.* Further studies are ongoing to uncover the precise mechanism through which these probiotics provide protection against *C. difficile*.

In our in-vivo study using mice, the newly developed human-origin probiotic cocktail ameliorated CDI, indicated by lower clinical scores, higher survival rates, reduced intestinal *C. difficile* burden and toxins in mice with probiotics treatment (Figure 2). Moreover, analysis based on tissues demonstrated that the probiotic cocktail strengthened the gut barrier, resulting in reduced gut permeability and gut inflammation (Figure 3). To assess how the probiotic cocktail improves gut health, gut microbiome and gut metabolites were analyzed. As expected, the probiotic cocktail increased the alpha diversity of the gut microbiome, improved the relative abundance of beneficial *Lactobacillus*, *Bifidobacterium*, and *Streptococcus* strains (Figure 4). In line with our study, Li et al. (2019) found that a probiotic mix of *Lactobacillus* and *Bifidobacterium* strains, isolated from human and animal sources, improved gut microbiota diversity and reduced *C. difficile* colonization in mice. While our high-diversity and high-target cocktail is non-invasive with probiotic strains exclusively isolated from human fecal samples, making them more suited to the human gut (Dogi and Perdigón, 2006; Russell et al., 2022; Zommiti et al., 2020). Worth noticing, the newly developed probiotic cocktail led to a decrease in the relative abundance of *Enterococcus* genera. As reported by Smith et al. (2022), Enterococci could enhance the fitness and pathogenesis of *C. difficile* (Smith et al., 2022). Inhibition of *Enterococcus* genera further enhances potential of Pro11 in combating CDI via beneficially modulating gut microbiome. Although Pro11 provide protection against the CDI, it comprises of 11 different bacterial strains, which could have differences in their action against *C. difficile.* So, further *in vitro* studies are being conducted to understand the effect of different combinations of the Pro11 strains to identify whether all the 11 probiotic strains are needed for protection and to understand the interaction between each strain. In our *in vivo* experiment, probiotics were added to the drinking water, which may not ensure that each mouse received the required dosage. Therefore, in future studies, including clinical trials, probiotics can be administered via oral gavage to ensure a precise and consistent dosage.

Along with modulation of gut microbiome, Pro11 also induced changes in gut metabolites with significantly different metabolites clusters with that from the control (Figure 5A). referring to specific metabolites, Pro11 increased 2-hydroxyisobutyrate, butyrate, glutamate, Sn-glycero-3-phosphcoline, leucine, and valine in the gut (Figure 5B). Metabolites like secondary bile acids and short-chain fatty acids (SCFAs) have been shown to inhibit *C. difficile* (Aguirre and Sorg, 2022; Łukawska and Mulak, 2022; Ouyang et al., 2022). Interestingly, among the major SCFAs, butyrate was significantly increased while acetate and propionate were decreased (Figure 5B). This is consistent with the report that butyrate rather than the other two, most consistently impact *C. difficile* fitness and be negatively associated with *C. difficile* burdens (Pensinger et al., 2023). Besides direct inhibition, butyrate can benefit the gut epithelium (Fachi et al., 2019; Hayashi et al., 2021; Łukawska and Mulak, 2022). Through the interaction with the host cells, butyrate can stimulate processes to improve the intestinal barrier and gut homeostasis (Nogal et al., 2021). A study by Wang et al. (2023) showed that butyrate could regulate the bile acid metabolism, strengthen the gut barrier and promote anti-inflammatory effects (Wang et al., 2023). In this study, among the top three probiotics-induced gut metabolites, glutamate could promote enterocyte proliferation, protect the intestinal mucosa, regulate tight junction proteins, suppress pro-inflammatory signaling pathways during normal and pathologic conditions (Kim and Kim, 2017). 2-Hydroxyisobutyrate (2HIB) was reported to extend life span, delay aging processes, and stimulate the oxidative stress resistance in nematodes (Schifano et al., 2022). To be noticed, glutamate can serve as precursor for butyrate production (Blachier et al., 2009; Buckel and Barker, 1974), and 2HIB can be synthesized from butyrate (Przybylski et al., 2013). Moreover, branched chain amino acids, like valine and leucine, are important for protein biosynthesis (Blomstrand et al., 2006). They are also found to play a role in strengthening the intestinal barrier via promoting the development of the epithelia cells, proliferation of enterocyte and enhance the immune response (Zhou et al., 2018). Increase of these beneficial gut metabolites are supposed to be involved in effects of the probiotic cocktail on protecting host against CDI. It will be interesting to further study the roles of these specific metabolites in *C. difficile* pathogenesis.

In conclusion, a human-origin highly diverse and highly targeted probiotic cocktail was developed in this study. The probiotic cocktail comprising 11 strains from *Lactobacillus, Bifidobacterium*, and *Streptococcus* protected mice against *C. difficile* infection by modulating gut microbiota and gut metabolites. Results from this study highlighted probiotics as a promising precise and sustainable approach against CDI. This study, along with further research into the underlying molecular mechanisms, safety across diverse populations, and scalability for large-scale production, will establish a solid foundation for translating probiotics into a clinical therapy for CDI. Additionally, these findings could inform similar investigations into other gastrointestinal diseases, such as inflammatory bowel diseases, where probiotics may enhance gut health and provide protective benefits.

## Data Availability

The datasets presented in this study can be found in online repositories. The names of the repository/repositories and accession number(s) can be found in the article/Supplementary material.
