# Supplementary material for: Non-invasive treatment of Clostridioides difficile infection with a human-origin probiotic cocktail through gut microbiome-gut metabolome modulations
Source: Front Microbiol. 2025 Feb 26;16:1555220. doi: 10.3389/fmicb.2025.1555220 (PMC11897039; doi:10.3389/fmicb.2025.1555220)
Supplement: Supplementary file 1 [file Data_Sheet_1.docx]

**Supplementary materials**

**Non-invasive treatment of *Clostridioides difficile* infection with a human-origin probiotic cocktail through gut microbiome-gut metabolome modulations**

Bijay Gurung^1,2^, Maria C Courreges^1^, Julie Pollak^3^, Ramiro Malgor^1^, Lin Jiang^4^, Bo Wang^3^, Shaohua Wang^1,2*^

^1^Department of Biomedical Sciences, Ohio University Heritage College of Osteopathic Medicine, Ohio University, Athens, OH, 45701 USA

^2^Infectious and Tropical Disease Institute, Ohio University, Athens, OH, 45701 USA

^3^Department of Biomedical and Chemical Engineering and Sciences, Florida Institute of Technology, Melbourne, Florida, 32901, USA.

^4^Division of Natural Sciences, New College of Florida, Sarasota, FL, 34243 USA

***Corresponding author:**

Shaohua Wang, Ph.D.,

Department of Biomedical Sciences, Ohio University Heritage College of Osteopathic Medicine, Ohio University

Address: 7 Depot St, Ohio University, Athens, OH, USA 45701

Email: [wangs4@ohio.edu](mailto:wangs4@ohio.edu)

Phone: 1-740-593-2355

Fax: 1-740-597-2778

**Supplementary figures**


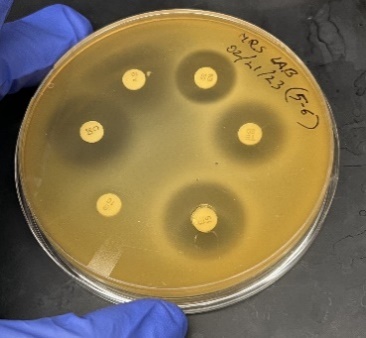


S

C

E

TE

NB

P

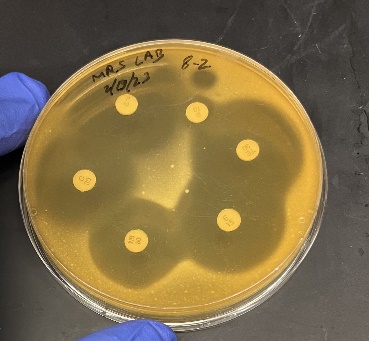


E

TE

S

C

NB

P

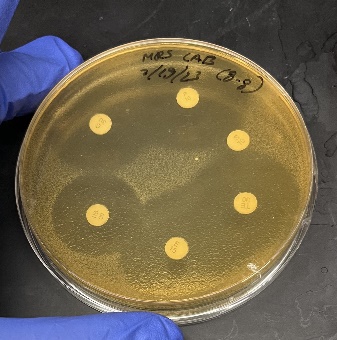


E

TE

S

P

C

NB


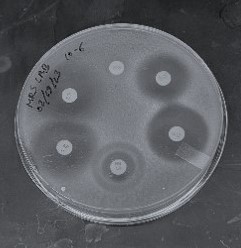


S

P

C

NB

E

TE

*LSW* 8-2

*LSW* 10-6

*LSW* 8-8

*BSW* 27-5

*BSW* 17-1


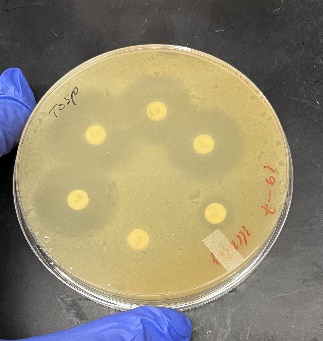


S

C

NB

E

P

TE


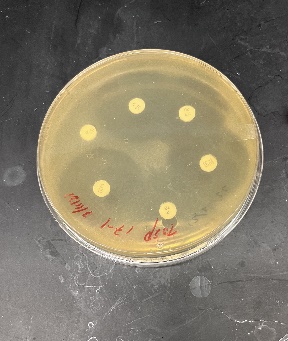


S

P

TE

C

NB

E


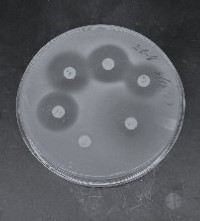


E

TE

P

S

NB

C

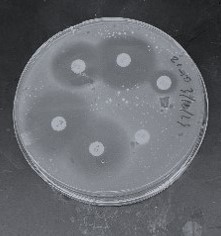


E

TE

NB

C

P

S


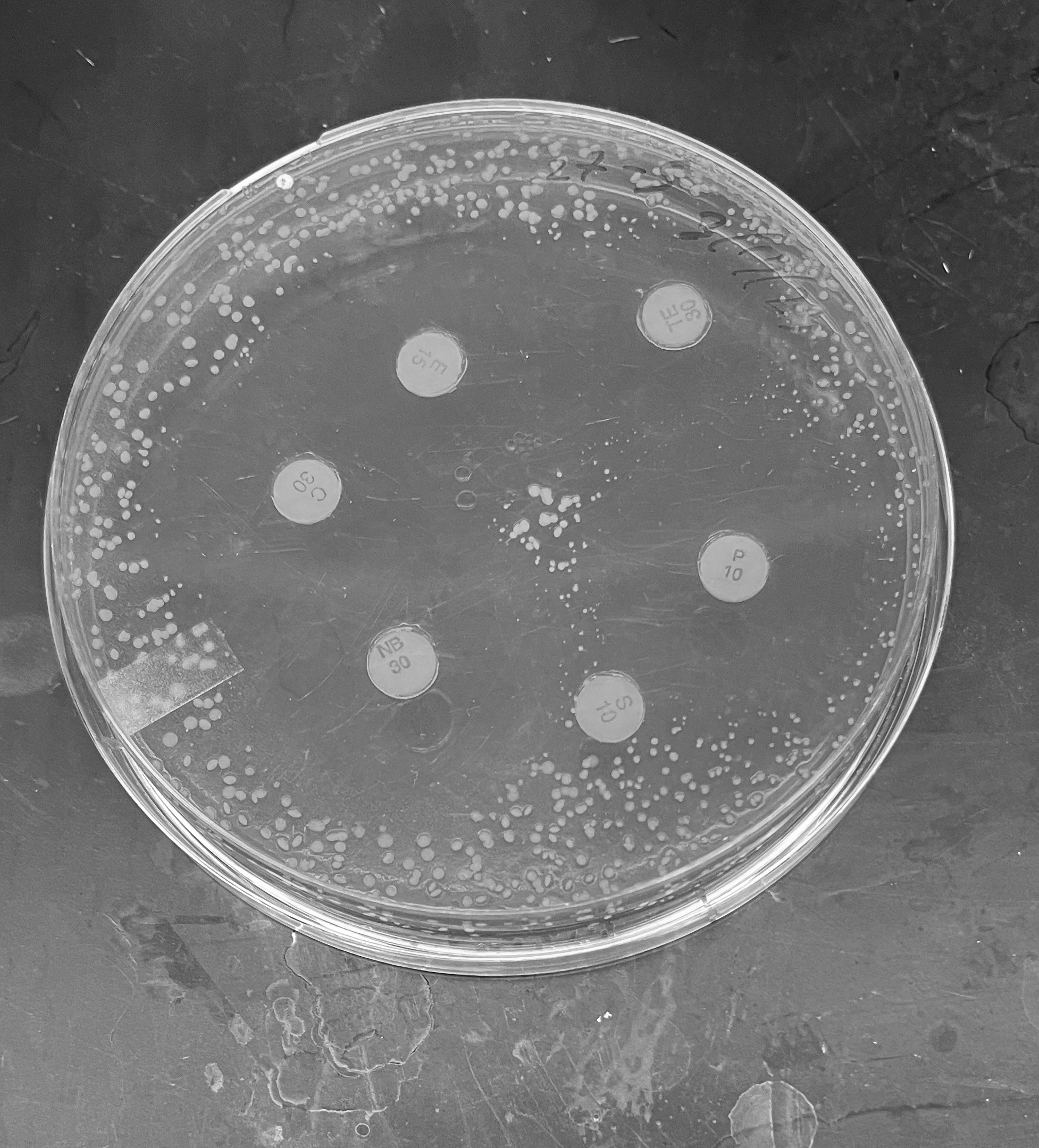


C

NB

S

P

TE

E

*BSW* 19-7

*BSW* 21-10

*BSW* 26-8


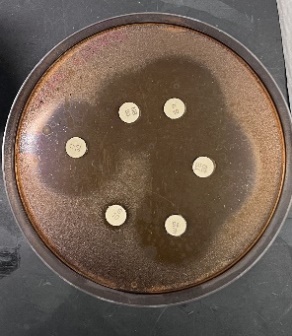


E

TE

S

P

NB

C

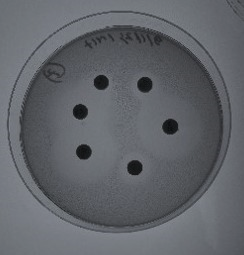


TE

S

E

C

NB

P

*SW* 17-11

*SW* 18-1

*LSW* 5-6

**Figure S1.** Antibiotic susceptibility test of the 11 probiotic strains. Antibiotics are S: Streptomycin, P: Penicillin, TE: Tetracycline, E: Erythromycin, NB: Novobiocin, C: Chloramphenicol.


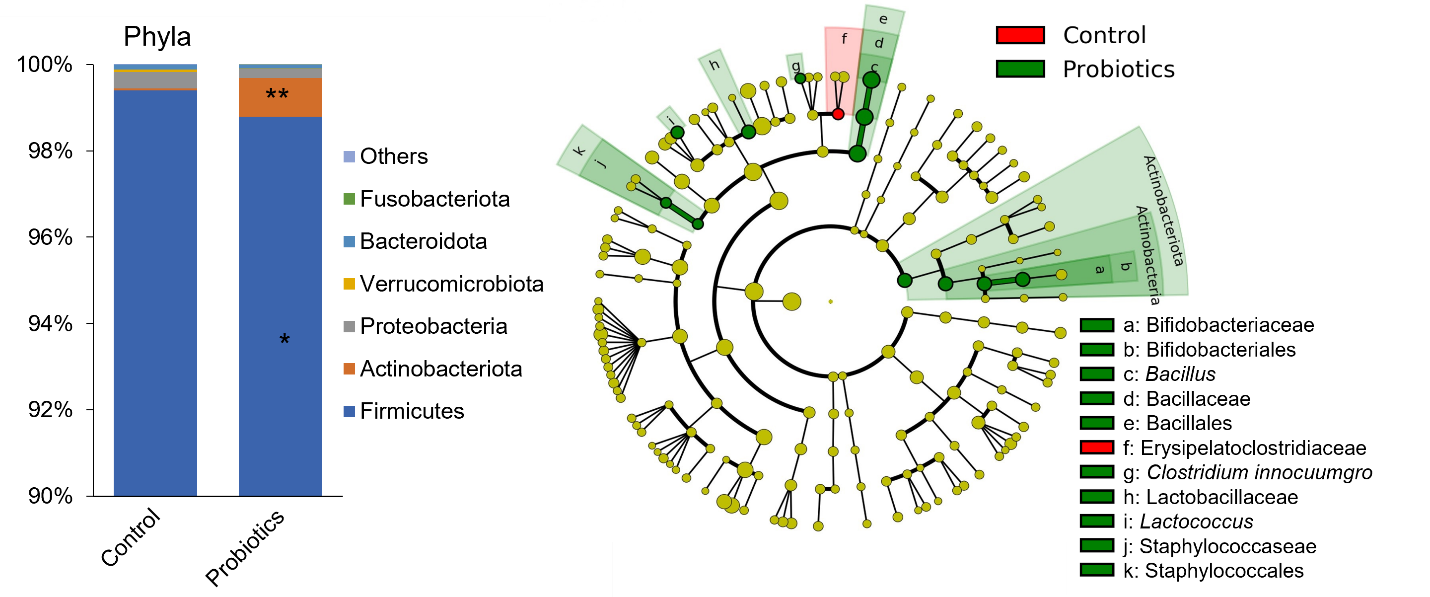


B

A

**Figure S2.** Effects of probiotic cocktail on gut microbiome modulation on phyla level (A) and demonstrated with Cladogram (B). P-values for differences between the probiotics-fed and control mice, * P<0.05, and ** p<0.01.
